# In Search of Preferential Macrocyclic Hosts for Sulfur Mustard Sensing and Recognition: A Computational Investigation through the New Composite Method r^2^SCAN-3c of the Key Factors Influencing the Host-Guest Interactions

**DOI:** 10.3390/nano12152517

**Published:** 2022-07-22

**Authors:** Fatine Ali Messiad, Nesrine Ammouchi, Youghourta Belhocine, Hanan Alhussain, Monira Galal Ghoniem, Ridha Ben Said, Fatima Adam Mohamed Ali, Seyfeddine Rahali

**Affiliations:** 1 Department of Process Engineering, Faculty of Technology, Université 20 Août 1955, El Hadaik Road, Skikda 21000, Algeria; f.alimessiad@univ-skikda.dz; 2 LRPCSI-Laboratoire de Recherche sur la Physico-Chimie des Surfaces et Interfaces, Université 20 Août 1955, Skikda 21000, Algeria; 3 Département de Technologie, Faculté de Technologie, Université 20 Août 1955, B.P. 26, Route d’El Hadaiek, Skikda 21000, Algeria; 4 Department of Chemistry, College of Science, Imam Mohammad Ibn Saud Islamic University (IMSIU), Riyadh 11432, Saudi Arabia; hmalhussain@imamu.edu.sa (H.A.); mgghoniem@imamu.edu.sa (M.G.G.); famohamedali@imamu.edu.sa (F.A.M.A.); 5 Department of Chemistry, College of Science and Arts, Qassim University, P.O. 53, Ar Rass 51921, Saudi Arabia; ben.said.ridha@gmail.com; 6 Laboratoire de Caractérisations, Applications et Modélisations des Matériaux, Faculté des Sciences de Tunis, Université Tunis El Manar, Tunis 2092, Tunisia

**Keywords:** non-covalent interactions, sulfur mustard, macrocycles, DFT-D4, inclusion complex, sensing

## Abstract

Sulfur mustard (SM) is a harmful warfare agent that poses a serious threat to human health and the environment. Thus, the design of porous materials capable of sensing and/or capturing SM is of utmost importance. In this paper, the interactions of SM and its derivatives with ethylpillar[5]arene (EtP[5]) and the interactions between SM and a variety of host macrocycles were investigated through molecular docking calculations and non-covalent interaction (NCI) analysis. The electronic quantum parameters were computed to assess the chemical sensing properties of the studied hosts toward SM. It was found that dispersion interactions contributed significantly to the overall complexation energy, leading to the stabilization of the investigated systems. DFT energy computations showed that SM was more efficiently complexed with DCMP[5] than the other hosts studied here. Furthermore, the studied macrocyclic containers could be used as host-based chemical sensors or receptors for SM. These findings could motivate experimenters to design efficient sensing and capturing materials for the detection of SM and its derivatives.

## 1. Introduction

Ethylene dichloride sulfide (bis(2-chloroethyl) sulfide) commonly known as sulfur mustard (SM) is a cytotoxic chemical compound with an oily liquid appearance belonging to the category of mass destruction weapons [1,2,3]. SM was first prepared in 1822 by César Despretz, however, it was not used until 1917 during the First World War [4]. SM is a highly toxic agent that attacks clammy skin, tissues, and airways, causing severe blisters and chemical burns to the eyes and mucous membranes, as well as long-term genetic damage [5,6]. In addition to its effects on health, SM is dangerous for the environment and can cause harmful effects on aquatic organisms. In this regard, addressing the problem of this chemical warfare agent and its derivatives is of particular importance.

Some decontamination processes have been developed to eliminate the SM such as chemical degradation by hydrolysis or oxidation [7,8]. An alternative strategy, based on the complexation of harmful substances [9,10,11,12] by macrocyclic systems offers new perspectives to address the issue of capturing and storing hazardous substances. Supramolecular chemistry is mainly concerned with the study of the host-guest complexes in which two or more neutral or charged molecules bind to each other via non-covalent interactions [13,14,15]; these interactions are the main driving forces of the complexation process, which leads to the modification of the physicochemical properties of host-guest complexes [16,17,18,19,20]. Among the supramolecular systems, cyclodextrins [21,22], cucurbiturils [23,24,25], calixarenes [26,27,28,29], and pillararenes [30,31,32] are the most studied host compounds. Moreover, self-assembled supramolecular architectures and nanostructures [33,34,35,36,37,38] play a key role in nanotechnologies and bioengineering.

**Figure 1:**
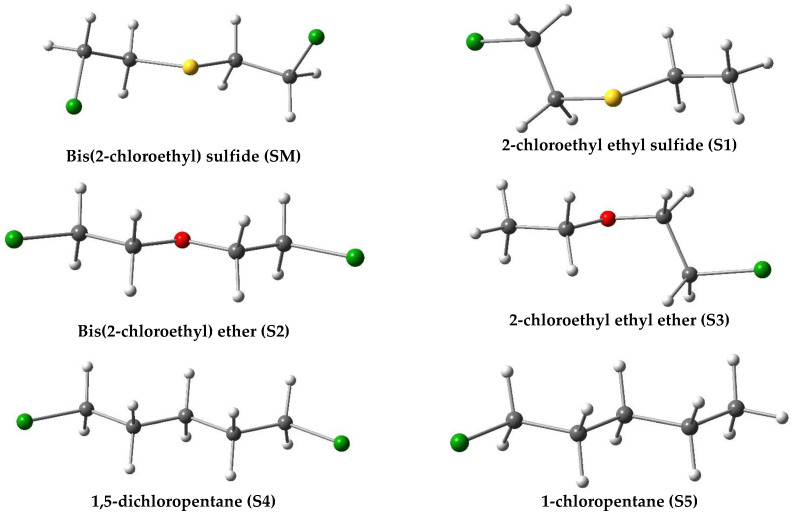
Optimized molecular structures of the guests: SM, S1, S2, S3, S4, and S5. Atom colors: chlorine (green); oxygen (red); carbon (grey); sulfur (yellow) and hydrogen (white).

In this context, Li et. Al. [39] conducted an experimental study in which per-ethylated pillar[5]arene (EtP[5]) host was used as a macrocyclic receptor for the recognition of the SM and its derivatives. The authors observed a strong binding and capture abilities toward SM and its stimulants with proven stability for a period of time (at least six months in the crystals) and discussed the mechanism of the interactions between (EtP[5]) and the guests SM, 2-chloroethyl ethyl sulfide (S1), bis(2-chloroethyl) ether (S2), 2-chloroethyl ethyl ether (S3), 1,5-dichloropentane (S4), and 1-chloropentane (S5). The optimized structures of SM and its stimulants (S1–S5) are represented in Figure 1.

From Figure 1, it is clear that S2 and S4 have a similar structure to that of SM, with the central sulfur atom replaced by oxygen or carbon atoms, respectively. The guests S3 and S5 are the monofunctional analogs of S2 and S4. X-ray diffraction showed that EtP[5] forms 1:1 inclusion complex with all the guests (SM-S5). The inclusion process is mainly driven by multiple C–H···π/Cl/S/O interactions. As a limitation of the study, the crystalline EtP[5] cannot be used for the degradation of SM. Therefore, it is interesting to functionalize the macrocyclic host systems as a strategy for the detoxification of SM [40], which may result in increasing the interaction energy and time storage.

Computational chemistry plays a crucial role in the field of host–guest complexation chemistry as an efficient tool for investigating the mechanism of the inclusion process [41,42,43,44] and for the prediction of new host molecules that can efficiently encapsulate the drug guests [45,46]. In this work, we present a DFT-D4 study of the host-guest interactions between the per-ethylated pillar[5]arene and SM and its simulants. Another important point of interest was to investigate the possible complexation of SM with several macrocyclic systems including functionalized pillar[5]arenes. Furthermore, different computational tools were used to analyze the structural, electronic, and sensing properties as well as the intermolecular interactions responsible for the stability of the formed complexes. We believe that this work will be useful for future experimental investigations aiming at capturing or sensing SM and its derivatives or analogs [47,48] using functionalized macrocyclic hosts.

## 2. Computational Methods

Full geometry optimization and energy calculations were performed using the recently developed meta-generalized-gradient approximation (mGGA) composite method r^2^SCAN-3c [49,50,51,52,53,54,55,56,57] combined with a modified version of the def2-TZVP basis set [58] denoted def2-mTZVPP [49]. A geometrical counterpoise correction (gCP) for the intra- and inter-molecular basis set superposition error was employed [59], as well as the Grimme dispersion term based on tight-binding partial charges (D4) [60,61,62] that was applied to account for the dispersion correction. All the DFT calculations were carried out with the ORCA program package (version 5.0.0) [63,64,65] in the gas phase. The complexation process between SM and its simulants and EtP[5] was also evaluated in o-xylene using the conductor-like polarizable continuum model (CPCM) [66,67]. The complexation energy (E_complexation_) for the SM and its derivatives with the studied macrocyclic hosts is computed by the following equation:

E_complexation_ = E_(complex)_ − (E_(host)_ + E_(guest)_) (1)

where E_(host)_ is the energy of the host molecule, E_(complex)_ is the energy of the host-guest complex, and E_(guest)_ is the energy of the guest molecule.

## 3. Results and Discussion

### 3.1. Interaction of SM and Its Simulants with EtP[5]

The starting geometries for the DFT geometry optimization of SM@EtP[5], S1@EtP[5], S2@EtP[5], S3@EtP[5], S4@EtP[5], and S5@EtP[5] complexes were retrieved from the crystal X-ray structures [39], corresponding, respectively, to the following assigned CCDC numbers 1831237 [68], 1884850 [69], 1831239 [70], 1884851 [71], 1831240 [72], and 1884852 [73].

#### 3.1.1. Structural and Energetic Properties

The optimized geometry and the values of the nearest intermolecular distances between SM and EtP[5] in SM@EtP[5] are visualized with Mercury 4.0 program [74] and presented in Table 1. The host-guest process is consisting of 1:1 ratio, in which non-covalent interactions play an important role in the stabilization of the formed complexes.

From a comparison of the experimental and optimized SM@EtP[5] complex geometries shown in Table 1, it is quite clear that the experimentally observed C–H···π/S/Cl intermolecular distances are reasonably reproduced in the gas phase by the r^2^SCAN-3c composite method. The sum of the deviations from the C–H…π experimental intermolecular distances noted ∆B in Table 1, obtained with r^2^SCAN-3c is 0.58 Å. The C–H···Cl and particularly the C–H···S hydrogen bond lengths are better predicted by r^2^SCAN-3c (∆B_C-H…Cl_ = 0.36 Å, ∆B_C-H…S_ = 0.21 Å).

In addition to the geometric parameters, the complexation energies were calculated with r^2^SCAN-3c in gas and o-xylene media (Table 2). The gas-phase dispersion-corrected energies were also evaluated and listed in Table 2.

Based on the association constant values of the six complexes determined by Li et al. [39], the ranking in the decreasing order follows the sequence K_a_ (S4) > K_a_ (SM) > K_a_ (S2) > K_a_ (S5) > K_a_ (S1) > K_a_ (S3). The same trend was observed (both in the gas phase and in o-xylene) by the calculated complexation energies with r^2^SCAN-3c except for S1@EtP[5] and S5@EtP[5]. Gas-phase complexation energies are −144.92, −141, −133.8, −122.74, −119.69, and −110.36 kJ/mol for S4@EtP[5], SM@EtP[5], S2@EtP[5], S1@EtP[5], S5@EtP[5], and S3@EtP[5], respectively. The computed complexation energies in o-xylene are found to be less negative. The results show that r_2_SCAN-3c calculations are in good agreement with the experimental association constants. From a structural and energetic point of view, the r^2^SCAN-3c describes well the systems studied in this work and will be therefore employed for subsequent calculations. The calculated dispersion energies with r^2^SCAN-3c functional in gas phase for SM@EtP[5], S1@EtP[5], S2@EtP[5], S3@EtP[5], S4@EtP[5], and S5@EtP[5] are, respectively, −55.48, −51.03, −50.31, −46.66, −53.72, and −50.09 kJ/mol (Table 2). The most stable complexes SM@EtP[5] and S4@EtP[5] have the highest dispersion energies of −55.48 and −53.72 kJ/mol whereas the less stable complex S3@EtP[5] has the lowest dispersion energy (−46.66 kJ/mol), indicating that dispersion interactions contribute significantly to the formation and stabilization of the complexes.

#### 3.1.2. Electronic Properties of SM, S1, S2, S3, S4, and S5@EtP[5] Complexes

For EtP[5] and all formed complexes, the chemical parameters such as frontier molecular orbitals (HOMO and LUMO), HOMO-LUMO energy gap [75,76], the percentage of HOMO–LUMO gap variation |ΔEg|% [77] and the dipole moment (µ) [78] were calculated in gas phase using r^2^SCAN-3c functional. The results are reported in Table 3.

As shown in Table 3, the HOMO, LUMO and HOMO-LUMO (H-L) gap energies are slightly varied upon the complexation of SM and its stimulants with EtP[5], however, the percentage of variation of HOMO–LUMO gap and the electric dipole moment of the two most stable complexes (SM@EtP[5] and S4@EtP[5]) are the highest among all complexes. The variation of |H-L| energy gap of SM@EtP[5] and S4@EtP[5] decreased, respectively, by 3.85 and 3.99 % after the complexation of SM and S4.

#### 3.1.3. NCI-RDG and IGMH Analysis of the Host-Guest Interactions

The identification of intra- and intermolecular interactions in supramolecular chemistry is important for quantifying the non-covalent forces responsible for the host-guest recognition [79].

Non-covalent interaction (NCI) analysis of reduced density gradient (RDG) [80] and independent gradient model based on Hirshfeld partition (IGMH) [81] can provide insights into the nature of host-guest interactions through the drawing of 3D color-filled isosurfaces representative of the occurring interactions, where blue, green, and red indicate, respectively, strong attractive interactions, van der Waals interactions and steric clashes.

**Figure 2:**
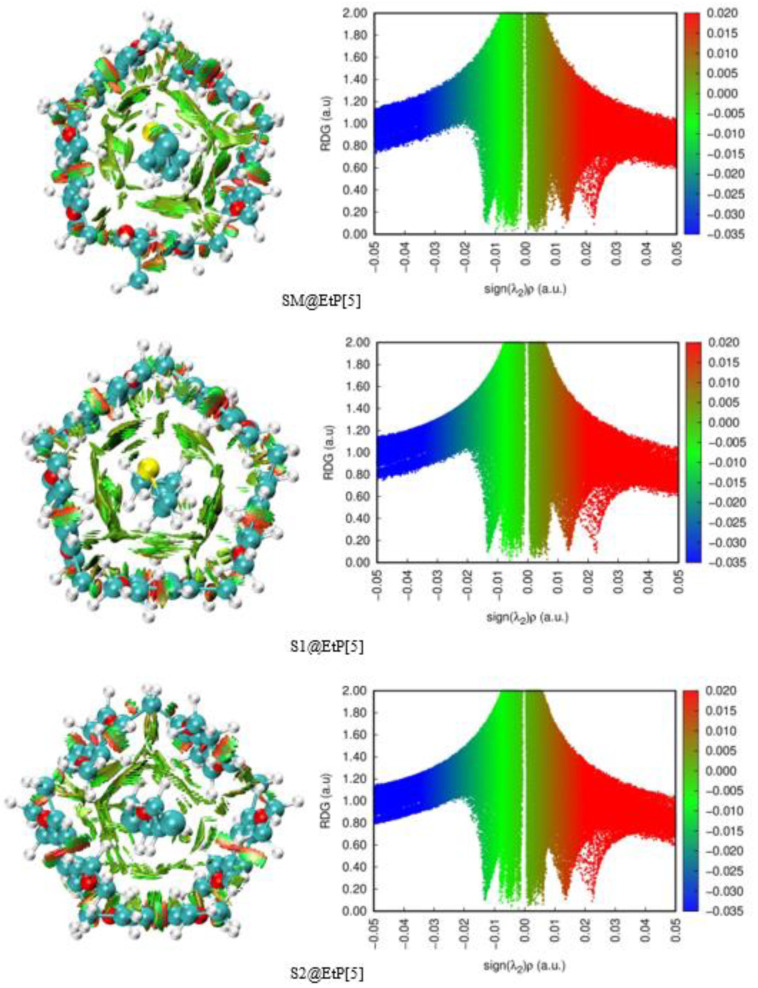

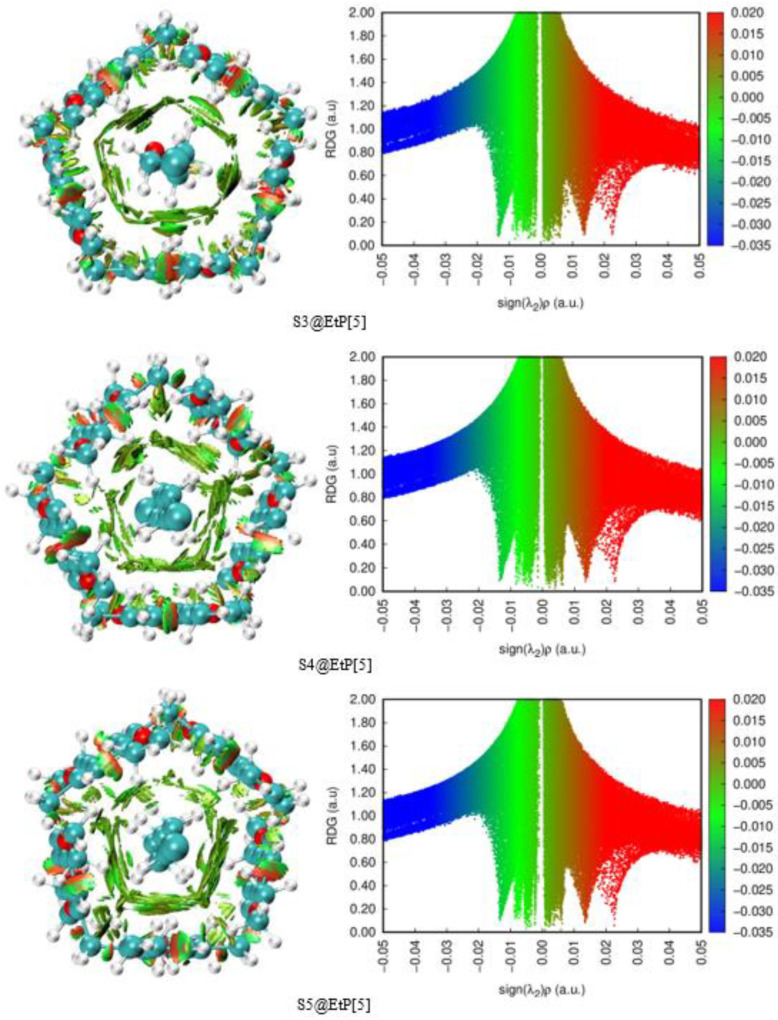
NCI-RDG isosurfaces (isovalue 0.5 a.u.) (**left**) and scatter plots (**right**) of SM@EtP[5], S1@EtP[5], S2@EtP[5], S3@EtP[5], S4@EtP[5], and S5@EtP[5].

The NCI-RDG isosurfaces (left figures of Figure 2) of all complexes and their scatter plots (right figures of Figure 2) were plotted with an isovalue of 0.5 a.u. in Figure 2. The NCI-RDG and IGM isosurfaces were visualized by VMD 1.9.3 program [82] through the outputs of Multiwfn 3.8 [83].

The visual characterization of NCI-RDG isosurfaces shows the presence of green-colored and light brown regions (left figures of Figure 2) in the interval [−0.015, 0.01 a.u.], indicating that weak dispersive forces are dominant in stabilizing the complex formation between SM and its stimulants, and the EtP[5] host. For SM@EtP[5], the peak occurring near −0.015 a.u. indicates mainly C-H···H-C and C-H···O intramolecular interactions, whereas the peaks in the range of −0.01 to 0.00 a.u. consist of mostly C-H···H-C, S···π, C-H···Cl, C-H···S, C-H···O and C-H···π intermolecular interactions. Similar conclusions are drawn using IGMH analysis (Supplementary data Figure S1).

DFT calculations show that the methylene groups are involved in the intermolecular interactions, indeed, the complex S4@EtP[5] with the highest number of methylene groups (Five CH_2_ groups) showed the highest complexation energy, followed by SM@EtP[5], S2@EtP[5], and S5@EtP[5] each with four CH_2_ groups and S1@EtP[5], and S3@EtP[5] complexes each with three CH_2_ groups. These findings suggest a strong correlation between C–H···π intermolecular interactions and the stability of the formed complexes [84]. The lower complexation energies of S2@EtP[5] and S3@EtP[5] in comparison, respectively, with SM@EtP[5], S4@EtP[5], and S1@EtP[5], S5@EtP[5] are due to the absence of intermolecular interactions between the central oxygen atom of S2 and S3 and the benzene moieties of EtP[5].

Due to their additive effect, the weak attractive C–H···π interactions play a key role as an important driving force in the inclusion process of SM and its derivatives within EtP[5].

DFT results indicated that weak hydrogen and halogen bonding (C–H···S, C–H···O and C–H···Cl) was overall observed in the studied complexes.

### 3.2. Interactions between SM and Different Macrocyclic Hosts

**Figure 3:**
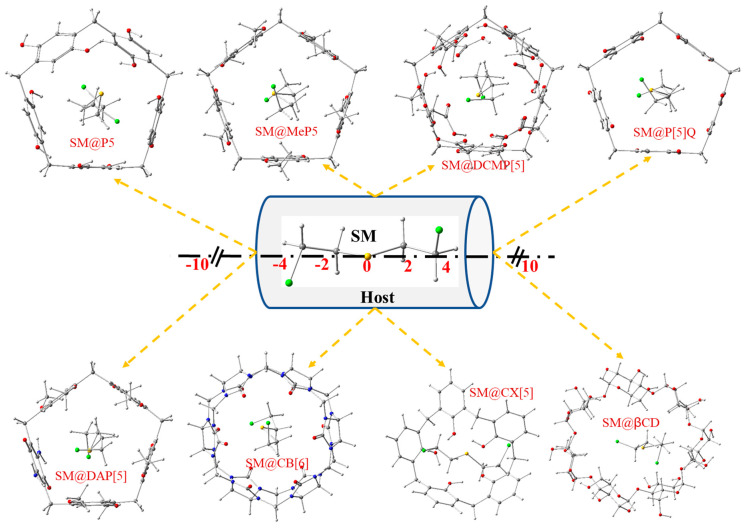
Coordinate systems of the complexation process of SM and eight macrocyclic molecules and the molecular structures of the most stable host-guest complexes. Atom colors: chlorine (green); oxygen (red); carbon (grey); sulfur (yellow); nitrogen (blue) and hydrogen (white).

The interaction of SM with eight macrocyclic hosts namely, pillar[5]arene (P[5]) [85], methylpillar[5]arene (MeP[5]) [32], Deca(carboxymethoxy)pillar[5]arene (DCMP[5]) [86,87,88], Pillar[5]quinone (P[5]Q) [89], mono(2,5-diamino-1,4-benzoquinone)pillar[5]arene (DAP[5]) [90], cucurbit[6]uril (CB[6]) [91], calix[5]arene (CX[5]) [92,93] and β-cyclodextrin (β-CD) [94,95] was studied computationally in gas phase using the r^2^SCAN-3c composite method. The most stable inclusion complexes were calculated based on the method elaborated by Liu and Guo [96]. The center of the guest and the host was set as the center of the coordination system, then SM was translated along the Z-axis from −10 to +10 Å with 2 Å step as shown in Figure 3. The complexation energies computed with r^2^SCAN-3c in gas phase as a function of the Z coordinate are displayed in Table 4. The optimized structures exhibit negative complexation energies, indicating an energetic favored process.

The most stable configurations for SM@P[5], SM@MeP[5], SM@DCMP[5], SM@P[5]Q, SM@DAP[5], SM@CB[6], SM@CX[5], and SM@β-CD with respective energies of −126.79, −113.16, −155.26, −90.77, −116.51, −96.94, −71.42 and −89.07 kJ/mol are located, respectively, at Z = −2 Å, Z = 0 Å, Z = 0 Å, Z = +8 Å, Z = −2 Å, Z = +4 Å, Z = +8 Å, and Z = +2 Å (Table 4). The geometries of the eight most stable complexes are visualized in Figure 3 using GaussView 5 software [97].

Among the studied hosts, the most important complexation energy was observed for DCMP[5] (−155.3 kJ/mol), which displays the highest complexation energy.

**Figure 4:**
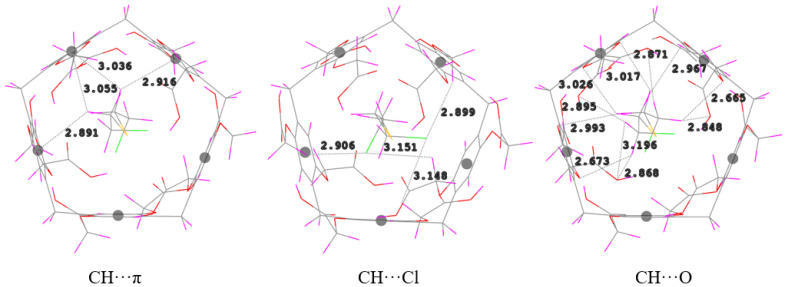
CH···π, CH···Cl and CH···O hydrogen bonding interactions in the complex SM@DCMP[5].

The SM guest is fully entrapped in the DCMP[5] cavity. The analysis of the nearest intermolecular distances occurring in the optimized structure of SM@DCMP[5] shows that there are four C–H···π interactions between the methylene groups of SM and the benzene moieties of DCMP[5], with distances ranging from 2.89 to 3.06 Å (Figure 4). Ea· chlorine atom of SM forms two C–H···Cl hydrogen bonds with the methylene moieties attached to the terminal -COOH groups of DCMP[5] at distances of 2.90 and 3.15 Å. Eleven C–H···O hydrogen bonds ranging from 2.67 to 3.20 Å were also observed between the –(CH_2_)– moieties of SM and the oxygen atoms of the –COOH groups of the host. However, no short C–H···S contacts were found between SM and DCMP[5].

#### 3.2.1. Electronic and Chemical Sensing Properties

Due to their efficiency and cost-effectiveness, macrocyclic host systems with cavities are relevant materials for sensing applications in medicine, biology, and environmental monitoring. The performance of such materials can be improved by introducing in their structures specific functional groups that enhance intermolecular interactions such as C–H···π, π–π, halogen and hydrogen bonding between the host and the guest molecules.

The host systems selected in this study have more functional groups and could exhibit improved sensing properties towards SM. The electronic quantum parameters such as HOMO and LUMO energies, HOMO–LUMO energy gap (|ΔE|_gap_), as well as the percentage of variation of HOMO–LUMO gap were calculated in the gas phase with the r^2^SCAN-3c composite method for the host molecules and their complexes with SM, are reported in Table 5.

The results of Table 5 show that after complexation of SM with host molecules, the |H-L| gap of SM@β-CD, SM@CB[6], SM@MeP[5], SM@CX[5] and SM@P[5]Q complexes decreases, respectively, from 6.11, 5.75, 3.52, 4.18, and 1.95 eV to 4.66, 3.98, 3.28, 3.58 and 0.95 eV, whereas it increases for SM@DCMP[5] and SM@DAP[5] complexes from 3.31 and 1.55 eV to 3.47 and 1.69 eV, respectively. However, the encapsulation of SM in P[5] does not affect the HOMO-LUMO gap energy.

The percentage variation in HOMO–LUMO gap |ΔEgap| upon the SM inclusion varies by 51.28, 30.78, 23.73, and 14.35 %, respectively, for SM@P[5]Q, SM@CB[6], SM@β-CD, and SM@CX[5] complexes, thus showing the potential of these macrocycles as a promising candidate for electronic sensing of SM.

**Figure 5:**
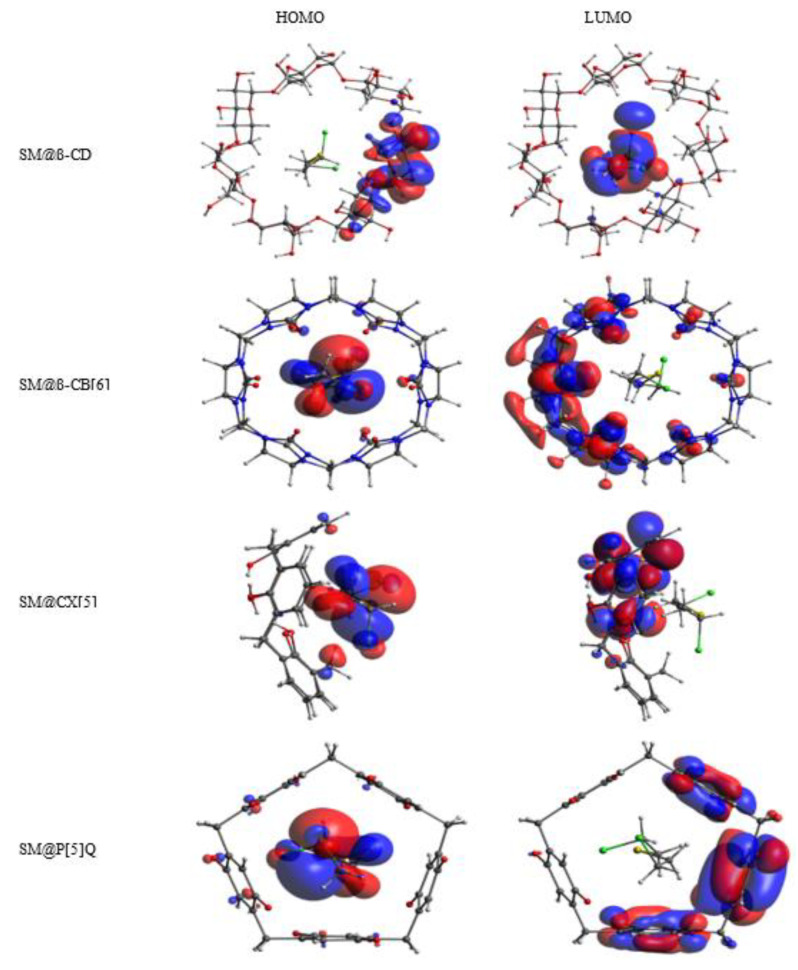
The frontier molecular orbitals (HOMO and LUMO) of SM@β-CD, SM@CB[6], SM@CX[5], and SM@P[5]Q obtained from r_2_SCAN-3c gas-phase calculations. Atom colors: chlorine (green); oxygen (red); carbon (grey); sulfur (yellow); nitrogen (blue) and hydrogen (white).

A pictorial representation of frontier molecular orbitals (HOMO and LUMO) of SM@β-CD, SM@CB[6], SM@CX[5] and SM@P[5]Q using AVOGADRO [98,99] is illustrated in Figure 5.

The HOMO and LUMO of SM@P[5], SM@DAP[5], SM@MeP[5], and SM@DCMP[5] are presented in Supplementary Figure S2.

The HOMO levels of SM@CB[6], SM@CX[5], and SM@P[5]Q complexes are mainly localized on SM and LUMO levels are localized on the host molecules (CB[6], CX[5], and P[5]Q), whereas the reverse is true for SM@β-CD complex. It is important to underline that the HOMO energies of SM@β-CD, SM@CB[6], SM@CX[5], and SM@P[5]Q increase from −6.14, −5.82, −5.44, and −6.68 eV to −5.98, −4.21, −5.14, and −5.88 eV and the LUMO energies decrease from −0.03, −0.07, −1.26, and −4.73 eV to −1.32, −0.23, −1.56, and −4.93 eV, respectively, this destabilization of HOMO energies and stabilization of LUMO energies leads to a significant reduction of the |H-L| gap and, therefore, the increase of the sensitivity and reactivity of β-CD, CB[6], CX[5] and P[5]Q hosts towards SM.

#### 3.2.2. NCI-RDG Analysis of SM@DCMP[5] Complex

**Figure 6:**
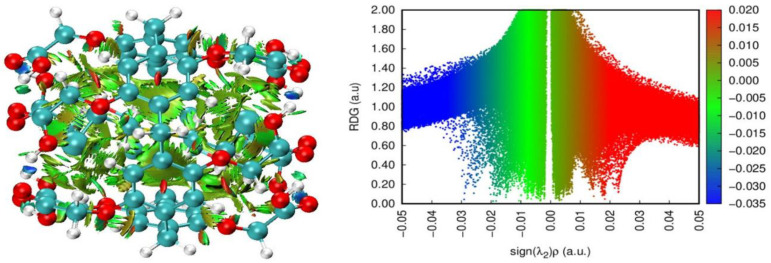
NCI-RDG isosurface (isovalue 0.5 a.u.) (**left**) and scatter plot (**right**) of SM@DCMP[5].

The results of NCI-RDG analysis show that in addition to the peaks (Figure 6) appearing between −0.01 and 0.00 a.u of C-H···H-C, S···π, C-H···Cl, C-H···O, and C-H···π intermolecular interactions, the complex SM@DCMP[5] exhibit mainly C-H···H-C, C-H···O-C, and C-H···O-H intramolecular interactions in the range (−0.03, −0.02 a.u.). The spikes appearing at ~−0.03 a.u. in Figure 6 (right) correspond to the intramolecular hydrogen bonds as revealed by the presence of four blue-colored disc-shaped isosurfaces (Figure 6-left) with O⋯H distances less than 2.0 Å (1.87, 1.87, 1.92, and 1.92 Å). Thus, these intramolecular hydrogen bonds contribute to stabilizing the complex SM@DCMP[5]. It is worth mentioning that the carboxyl end groups are remarkably pointing to the interior of DCMP[5] cavity upon the SM inclusion, allowing, therefore, the formation of a circular intramolecular hydrogen-bond network. Moreover, the SM is totally sequestered in the cavity of DCMP[5].

## 4. Conclusions

The present investigation aimed at providing an insight into the in-depth understanding of the interactions governing the structure and host-guest complexation of sulfur mustard and its derivatives with different macrocyclic systems using the newly developed composite method r^2^SCAN-3c. The analysis of the obtained results comes to the following conclusions:

- The r^2^SCAN-3c method can reproduce satisfactorily the crystalline structures of SM@EtP[5], S1@EtP[5], S2@EtP[5], S3@EtP[5], S4@EtP[5], and S5@EtP[5] complexes.
- The complexation energies calculated using r^2^SCAN-3c correlate with the experimental association constants.
- The major forces that contribute to the stability of the formed complexes involve C-H···H-C, C-H···O intramolecular interactions and C-H···H-C, S···π, C-H···Cl, C-H···S, C-H···O and C-H···π intermolecular interactions as revealed by NCI-RDG and IGM analysis.
- The macrocycles CB[6], β-CD, CX[5] and particularly P[5]Q show great potential as sensors for sulfur mustard.
- Among the studied complexes, SM@DCMP[5] was the most stable with the highest complexation energy of −155.26 kJ/mol, its high stability is due to the occurrence of additional intramolecular hydrogen bonds in DCMP[5].

## Figures and Tables

**Table 1:** Experimental and computed nearest intermolecular distances (Å) of SM@EtP[5] complex. (The values in brackets represent the difference between the computed and the experimental distances).

Interaction	Interaction Distance	r^2^SCAN-3c	Exp. [39]	Gas-Phase Optimized Geometries
C–H···π	B_a_	2.82 (0.01)	2.81	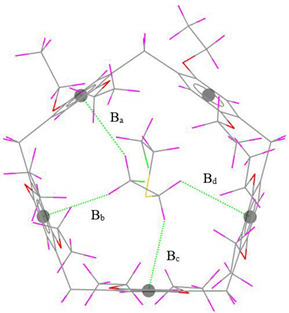
	B_b_	2.55 (0.13)	2.68	
	B_c_	2.56 (0.18)	2.74	
	B_d_	2.70 (0.26)	2.96	
	∆B	0.58	-	
C–H···Cl	B_e_	3.02 (0.20)	3.22	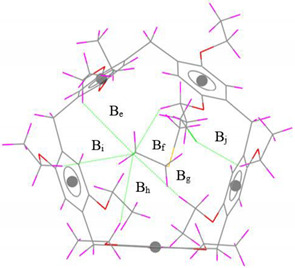
	B_f_	3.05 (0.03)	3.08	
	B_g_	3.35 (0.01)	3.34	
	B_h_	3.14 (0.06)	3.20	
	B_i_	3.19 (0.04)	3.15	
	B_j_	3.34 (0.02)	(3.32)	
	∆B	0.36	-	
C–H···S	B_k_	3.24 (0.08)	3.32	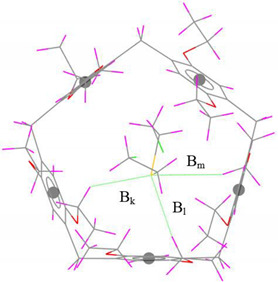
	B_l_	3.01 (0.11)	3.12	
	B_m_	3.18 (0.02)	3.16	
	∆B	0.21	-	

**Table 2:** Calculated complexation and dispersion-corrected energies (kJ/mol) and experimental values of association constants in M^−1^.

Complex	r^2^SCAN-3c (Gas Phase)	r^2^SCAN-3c (O-Xylene)	r^2^SCAN-3c Gas Phase Dispersion Energy	Association Constants (M^−1^) [39]
SM@EtP[5]	−141	−114.44	−55.48	6.2 × 10^3^
S1@EtP[5]	−122.74	−105.69	−51.03	2.9 × 10^2^
S2@EtP[5]	−133.76	−113.01	−50.31	1.3 × 10^3^
S3@EtP[5]	−110.36	−97.43	−46.66	67
S4@EtP[5]	−144.92	−118.35	−53.72	1.8 × 10^4^
S5@EtP[5]	−119.69	−103.09	−50.09	7.9 × 10^2^

**Table 3:** Calculated chemical parameters for EtP[5] and all complexes using r^2^SCAN-3c in the gas phase.

Complex	HOMO (eV)	LUMO (eV)	|H-L| gap (eV)	|ΔEg| %	µ (Debye)
EtP[5]	−4.38	−0.87	3.51	-	0.06
SM@EtP[5]	−4.44	−1.06	3.38	3.85	3.44
S1@EtP[5]	−4.43	−0.95	3.48	0.84	1.87
S2@EtP[5]	−4.47	−1.01	3.46	1.42	0.88
S3@EtP[5]	−4.41	−0.97	3.44	1.99	1.87
S4@EtP[5]	−4.44	−1.07	3.37	3.99	2.11
S5@EtP[5]	−4.43	−0.98	3.45	1.71	1.82

**Table 4:** Complexation energies (kJ/mol) between SM and the eight studied macrocycles calculated with r^2^SCAN-3c in the gas phase.

Position (Å)	SM@P[5]	SM@MeP[5]	SM@DCMP[5]	SM@P[5]Q	SM@DAP[5]	SM@CB[6]	SM@CX[5]	SM@β-CD
−10	−54.25	−29.41	−83.32	−90.51	−44.74	−72.12	−32.35	−56.82
−8	−77.19	−109.80	−121.57	−74.96	−71.12	−68.88	−32.77	−52.10
−6	−77.28	−110.00	−121.66	−90.53	−114.52	−75.41	−51.72	−51.61
−4	−123.65	−109.99	−118.70	−87.29	−112.35	−96.42	-	−54.82
−2	−126.79	−110.00	−120.90	−85.67	−116.51	−96.44	-	−55.36
0	−123.62	−113.16	−155.26	−86.63	−112.37	−96.80	-	−79.49
+2	−101.89	−109.90	−128.54	−90.23	−114.22	−95.76	-	−89.07
+4	−101.97	−109.92	−118.63	−90.38	−114.18	−96.94	-	−87.11
+6	−101.67	−109.93	−122.04	−90.33	−114.32	−75.28	−71.41	−85.58
+8	−99.42	−109.66	−80.91	−90.77	−37.20	−69.12	−71.42	−81.01
+10	−99.40	−29.50	−85.44	−90.69	−96.49	−73.44	−53.95	−78.78

**Table 5:** Calculated HOMO, LUMO, HOMO-LUMO energy gap and the percentage of variation of HOMO–LUMO gap of studied complexes with r^2^SCAN-3c in gas phase.

Host/Complex	Electronic Chemical Parameters			
	E_HOMO_ (eV)	E_LUMO_ (eV)	|ΔE|_gap_ (eV)	∆E_g %_
β-CD	−6.14	−0.03	6.11	23.73
SM@β-CD	−5.98	−1.32	4.66	
CB[6]	−5.82	−0.07	5.75	30.78
SM@CB[6]	−4.21	−0.23	3.98	
P[5]	−4.54	−1.49	3.05	0.00
SM@P[5]	−4.70	−1.65	3.05	
MeP[5]	−4.44	−0.92	3.52	6.82
SM@MeP[5]	−4.40	−1.12	3.28	
DCMP[5]	−5.10	−1.79	3.31	4.83
SM@DCMP[5]	−5.23	−1.76	3.47	
DAP[5]	−4.56	−3.01	1.55	9.03
SM@DAP[5]	−4.56	−2.87	1.69	
CX[5]	−5.44	−1.26	4.18	14.35
SM@CX[5]	−5.14	−1.56	3.58	
P[5]Q	−6.68	−4.73	1.95	51.28
SM@P[5]Q	−5.88	−4.93	0.95	

## Data Availability

The data presented in this study are available on request from the corresponding author.

## Supplementary Materials

The following supporting information can be downloaded at: https://www.mdpi.com/article/10.3390/nano12152517/s1, Figure S1: IGMH isosurfaces (isovalue 0.007 a.u.) (upper figures) and scatter plots (lower figures) of SM@EtP[5], S1@EtP[5], S2@EtP[5], S3@EtP[5], S4@EtP[5] and S5@EtP[5]; Figure S2: The frontier molecular orbitals (HOMO and LUMO) of SM@P[5], SM@DAP[5], SM@MeP[5] and SM@DCMP[5] obtained from r^2^SCAN-3c gas phase calculations.
