# Supplementary material for: In Search of Preferential Macrocyclic Hosts for Sulfur Mustard Sensing and Recognition: A Computational Investigation through the New Composite Method r2SCAN-3c of the Key Factors Influencing the Host-Guest Interactions
Source: Nanomaterials (Basel). 2022 Jul 22;12(15):2517. doi: 10.3390/nano12152517 (PMC9329917; doi:10.3390/nano12152517)
Supplement: Supplementary file 1 [file nanomaterials-12-02517-s001.zip › nanomaterials-1817940-supplementary.pdf]

In Search of Preferential Macrocyclic Hosts for Sulfur Mustard Sensing and Recognition: A Computational Investigation through the New Composite Method r^2SCAN-3c of the Key Factors Influencing the Host-Guest Interactions

Fatine Ali Messiad [1,2], Nesrine Ammouchi [2,3,*], Youghourta Belhocine [1,*], Hanan Alhussain [4], Monira Galal Ghoniem [4], Ridha Ben Said [5,6], Fatima Adam Mohamed Ali [4] and Seyfeddine Rahali [5,*]

[1] Department of Process Engineering, Faculty of Technology, 20 August 1955 University of Skikda, El Hadaik Road, Skikda 21000, Algeria; f.alimessiad@univ-skikda.dz
[2] LRPCSI-Laboratoire de Recherche sur la Physico-Chimie des Surfaces et Interfaces, Université 20 Août 1955-Skikda, Skikda 21000, Algeria
[3] Département de Technologie, Faculté de Technologie, Université 20 Août 1955, B.P. 26, Route d'El Hadaiek, Skikda 21000, Algeria
[4] Department of Chemistry, College of Science, Imam Mohammad Ibn Saud Islamic University (IMSIU),
Riyadh 11432, Saudi Arabia; hmalhussain@imamu.edu.sa (H.A.);
mgghoniem@imamu.edu.sa (M.G.G.)
famohamedali@imamu.edu.sa (F.A.M.A.)
[5] Department of Chemistry, College of Science and Arts, Qassim University, P.O. 53, 51921 Ar Rass, Saudi Arabia; ben.said.ridha@gmail.com
[6] Laboratoire de Caractérisations, Applications et Modélisations des Matériaux, Faculté des Sciences de
Tunis, Université Tunis El Manar, 2092 Tunis, Tunisie
* Correspondence: n.ammouchi@univ-skikda.dz (N.A.); y.belhocine@univ-skikda.dz (Y.B.);
saif.rahali@gmail.com (S.R.)

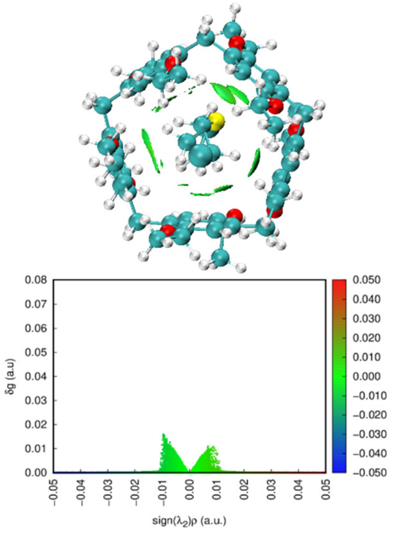

SM@EtP[5

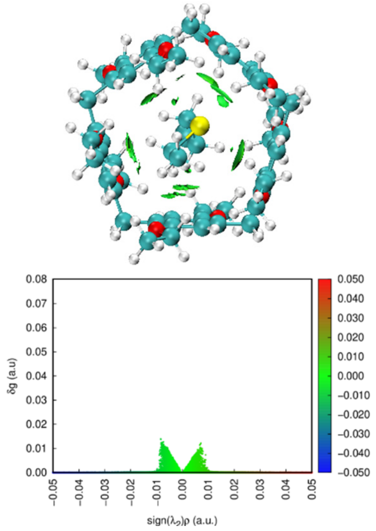

S1@EtP[5]

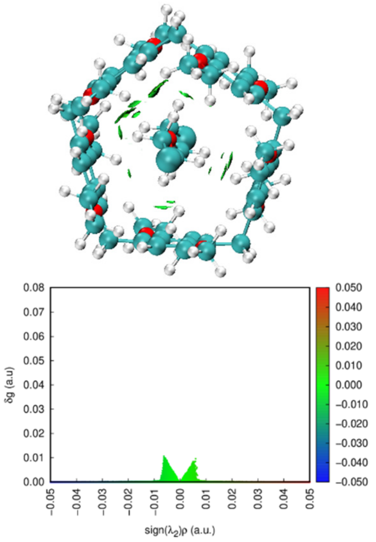

S2@EtP[5]

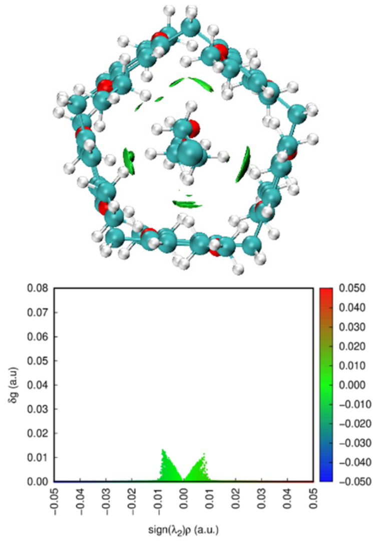

S3@EtP[5]

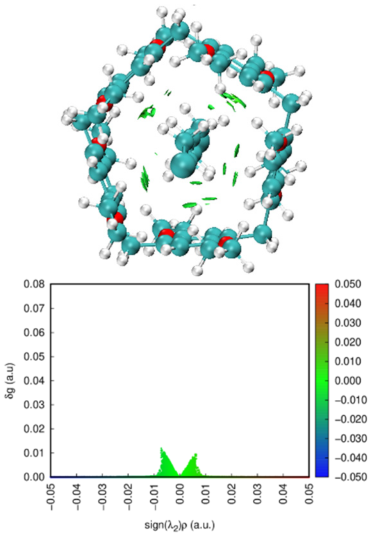

S4@EtP[5]

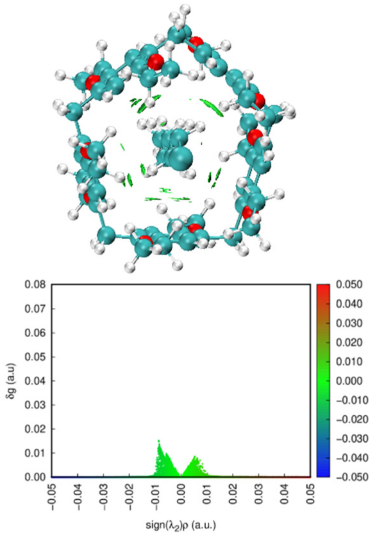

S5@EtP[5]

Figure S1. IGMH isosurfaces (isovalue 0.007 a.u.) (upper figures) and scatter plots (lower figures) of SM@EtP[5], S1@EtP[5], S2@EtP[5], S3@EtP[5], S4@EtP[5] and S5@EtP[5].

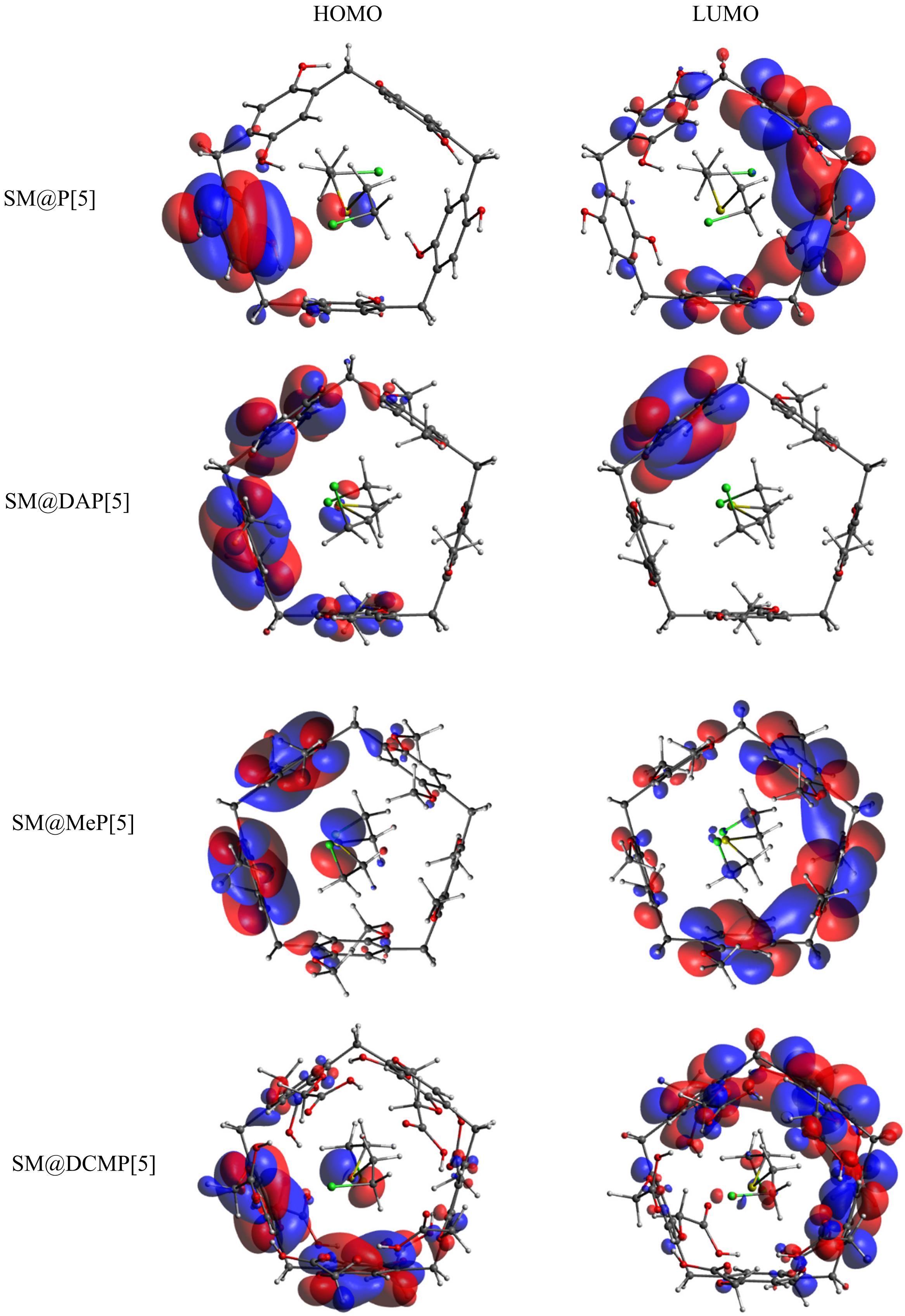

Figure S2. The frontier molecular orbitals (HOMO and LUMO) of SM@P[5], SM@DAP[5], SM@MeP[5] and SM@DCMP[5] obtained from r^2SCAN-3c gas phase calculations
